# Application of continuous nursing care based on hierarchical diagnosis and treatment mode in Stage II cardiac rehabilitation of patients after percutaneous coronary intervention

**DOI:** 10.3389/fcvm.2022.922449

**Published:** 2022-10-18

**Authors:** Zhen-Juan Dai, Jian-Ying Zhou, Shen-Ting Xu, Jian-Feng Zhang, Cui-Fang Zhuang, Pin-Hua Gu

**Affiliations:** ^1^Department of Nursing Care, Shanghai Songjiang District Central Hospital, Shanghai, China; ^2^Department of Rehabilitation, Shanghai Songjiang District Central Hospital, Shanghai, China

**Keywords:** cardiac rehabilitation, hierarchical diagnosis and treatment, continuous nursing care, percutaneous coronary intervention (PCI), Stage II cardiac rehabilitation

## Abstract

**Objective:**

To explore the effect of applying continuous nursing care based on hierarchical diagnosis and treatment in Stage II cardiac rehabilitation of patients after percutaneous coronary intervention (PCI) and provide a theoretical basis for clinical nursing intervention.

**Methods:**

Patients in PCI postoperative II cardiac rehabilitation were selected and randomly divided into the experimental group (community group), the experimental group (rehabilitation group), and the control group. Patients in the control group received the routine cardiac rehabilitation management scheme, while those in the experimental groups received continuous nursing protocol based on the hierarchical diagnosis and treatment mode. All patients were compared for the cardiac rehabilitation evaluation indexes at discharge and 6 months after discharge.

**Results:**

Compared with the control group, the left ventricular ejection fraction, 6-minute walking distance test, medication compliance, and quality of life were all improved in the two experimental groups, and the differences were statistically significant (*P* < 0.05). The improvement in the rehabilitation group was more significant than in the community group. There were more patients with high cardiac rehabilitation compliance in the rehabilitation group than in the community group, with the difference being statistically significant (*P* < 0.05).

**Conclusion:**

Continuous nursing care rehabilitation based on the hierarchical diagnosis and treatment mode can improve the cardiac function of patients after PCI, enhance their quality of life, and improve their rehabilitation and medication compliance, facilitating their cardiac rehabilitation.

## Introduction

According to the *Report on Cardiovascular Health and Diseases in China 2019* (1), there are about 330 million cases of cardiovascular diseases in China, and the prevalence rate continues to rise. As stated in the report, two out of every five deaths are due to cardiovascular disease, with the mortality staying at a high level. Clinical studies have demonstrated that cardiac rehabilitation can reduce the all-cause mortality of patients after myocardial infarction by 8–37% and the cardiovascular mortality by 7–38% through the combined action of five major prescriptions. As shown in the *Guidelines for Cardiac Rehabilitation and Secondary Prevention in China* (GCRSPC) (2), Stage II cardiac rehabilitation is generally carried out within 1–6 months after discharge. With the maturity of percutaneous coronary intervention (PCI) technology, the postoperative length of stay of patients is significantly shortened. Stage I rehabilitation time is limited; Stage III rehabilitation is mainly to maintain the health and exercise habits formed in the previous two stages; and Stage II, which involves early rehabilitation outside the hospital, is particularly important (3). This study aimed to explore the application of continuous nursing care in Stage II cardiac rehabilitation of patients after PCI under the background of the hierarchical diagnosis and treatment mode to provide a reference path for the implementation of cardiac rehabilitation.

## Materials and methods

### Demographics

A total of 100 patients discharged from Shanghai Songjiang District Central Hospital after initial PCI from July 2021 to December 2021 were randomly selected as the study subjects.

The inclusion criteria were as follows: (1) patients who were definitively diagnosed as coronary heart disease according to the World Health Organization diagnostic criteria; (2) patients who were permanent residents of the Songjiang District of Shanghai who underwent PCI during hospitalization; (3) patients whose risk stratification complied with low–medium risk of coronary heart disease (CHD) in accordance with the GCRSPC; (4) patients with <30% residual stenosis after PCI as shown by coronary angiography; (5) patients who voluntarily participated in the study and signed the informed consent; and (6) patients aged 18–75 years.

The exclusion criteria were as follows: (1) patients complicated with aortic stenosis, valvular disease, or hypertrophic obstructive cardiomyopathy; (2) patients complicated with tumors, thrombosis, severe hepatic and renal insufficiency, or other diseases that may aggravate the condition; (3) patients with postoperative complications, such as bleeding, embolism, restenosis, or occlusion; (4) patients classified as high risk according to the *Risk Stratification of Patients with Coronary Heart Disease*; (5) patients who did not live in the region in the recent period (within half a year); and (6) patients with loss of consciousness, mental illness, physical dysfunction, disability, or who were unable to cooperate with rehabilitation.

The elimination criteria were as follows: (1) patients with coronary heart disease risk stratification from low–medium risk to high risk; and (2) patients who died or dropped out of the study. Two patients who failed to meet the criteria and seven patients who were absent from data collection or withdrew from the study were excluded.

### Demographics of patients in the three groups

There were no statistically significant differences in age, gender ratio, left ventricular ejection fraction (LVEF) (%), the 6-minute walking distance (6MWD) test (m) and BMI of patients among the three groups (*P* > 0.05, Table 1).

**Table 1:** Demographics of patients in three groups ($\bar{x}$ ± s).

**Group**	**Number of cases**	**Age**	**Gender**	**LVEF**	**6MWD**	**BMI**
		**(years)**	**(male/female)**	**(%)**	**(m)**	**(kg /m^2^)**
Control group	28	67 (7.5)	15/12	47.86 ± 5.18	322.21 ± 50.75	25.49 ± 1.44
Community group	30	64 (11.25)	17/13	49.20 ± 4.80	317.3 ± 63.17	25.57 ± 1.59
Rehabilitation group	33	65 (9)	18/15	48.20 ± 5.23	303.33 ± 61.31	26.22 ± 1.49
*F*/X^2^		4.568	0.029	0.842	0.859	2.256
*P*		0.102	0.972	0.434	0.427	0.111

LVEF, Left ventricular ejection fraction; 6MWD, 6-minute walking distance; BMI, Body Mass Index.

### Methods

#### Intervention methods

##### Control group

Patients were given routine nursing care. Five major prescription manuals were issued according to the GCRSPC 2018 Edition. After discharge, they went home directly and were followed up at 1 month, 3 months, and 6 months.

##### Experimental group (community group)

**Figure 1:**
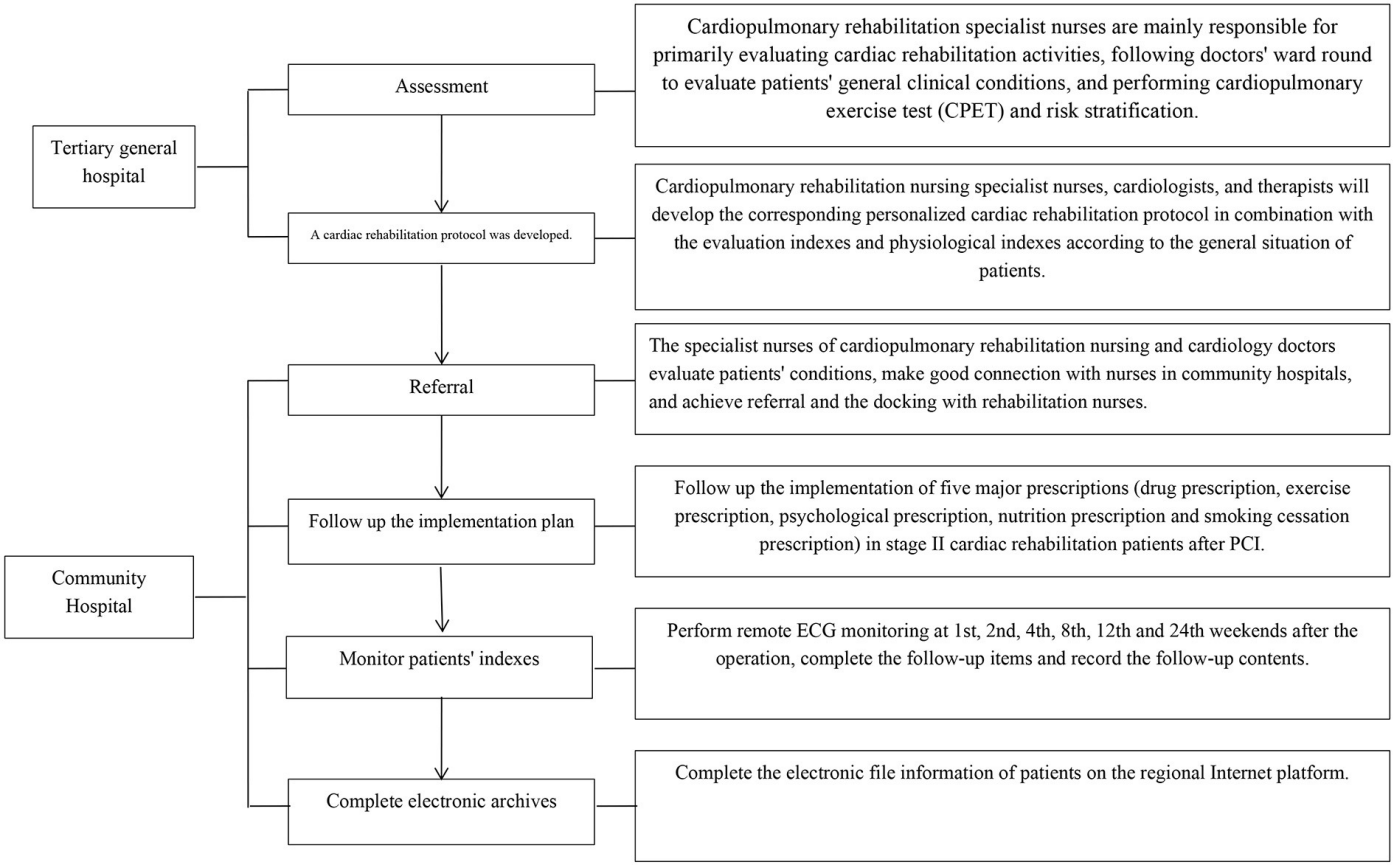
Continuous nursing cardiac rehabilitation mode based on hierarchical diagnosis and treatment-Tertiary General Hospital-Community Hospital.

Patients were given continuous nursing care based on the hierarchical diagnosis and treatment mode and were transferred to a community hospital for subsequent Stage II cardiac rehabilitation after discharge, as shown in Figure 1.

##### Experimental group (rehabilitation group)

**Figure 2:**
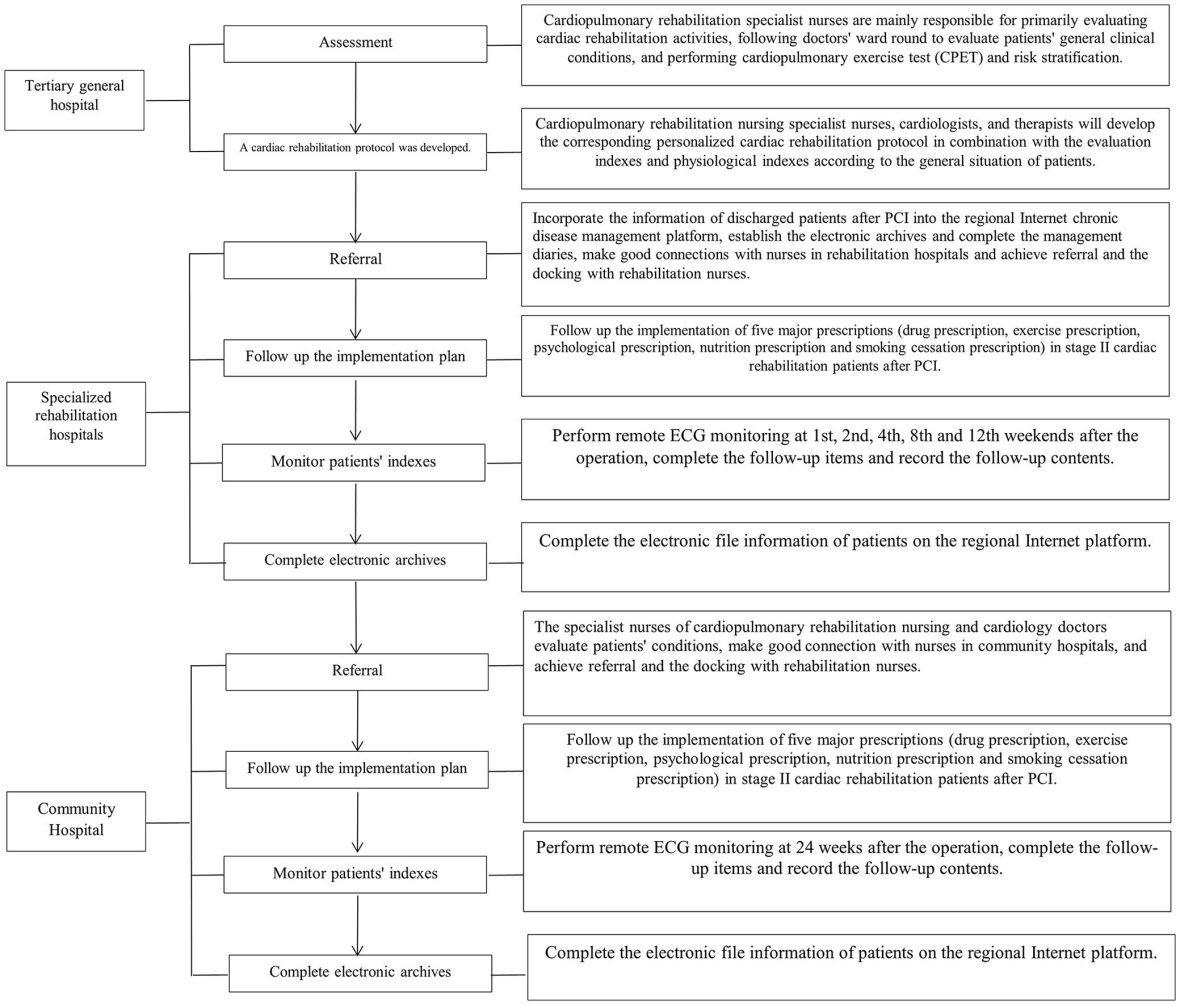
Continuous nursing cardiac rehabilitation mode based on hierarchical diagnosis and treatment-Tertiary General Hospital-Specialized Rehabilitation Hospital-Community Hospital.

**Figure 3:**
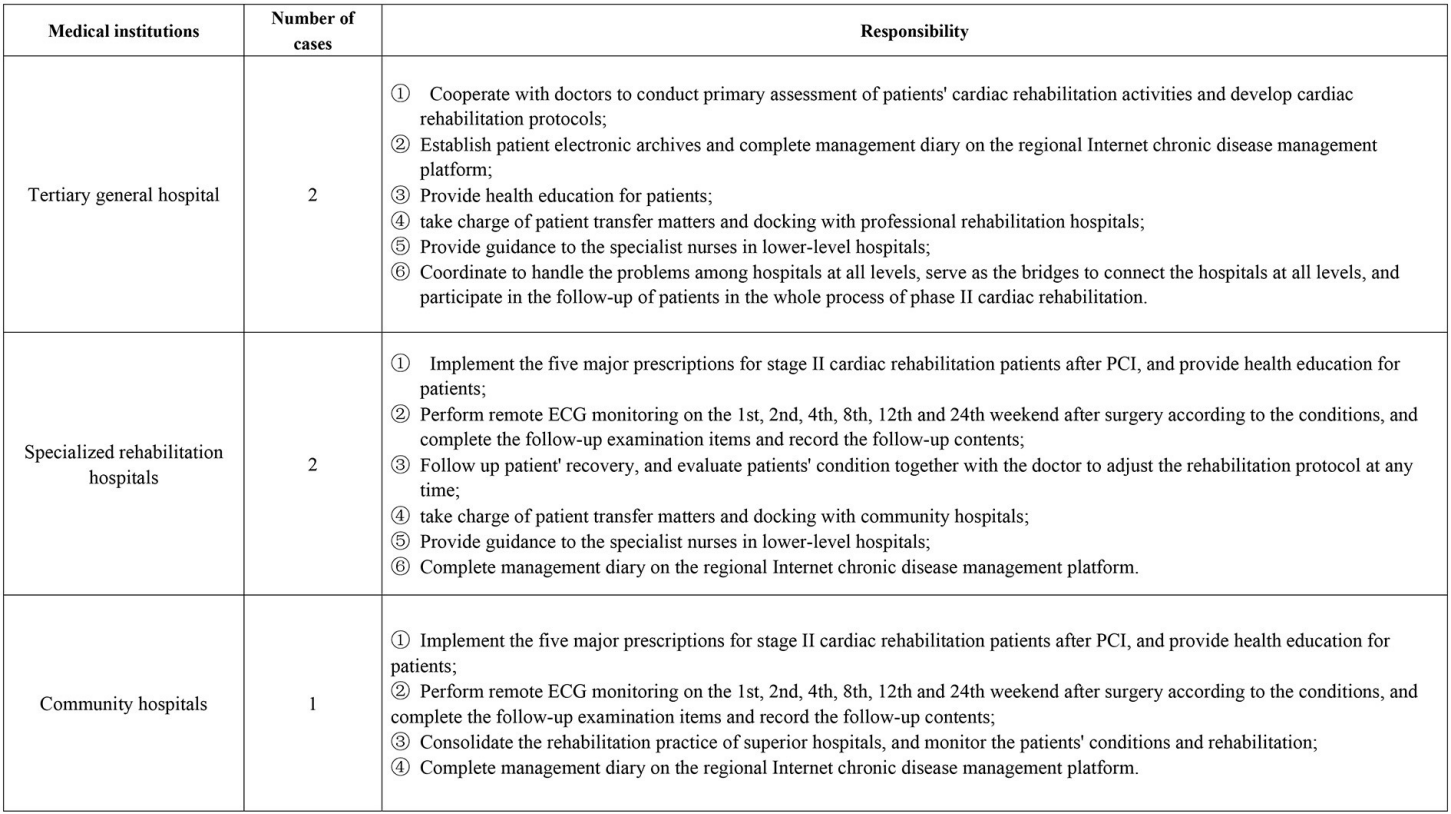
Responsibilities of specialist nurses of cardiopulmonary rehabilitation nursing care. Cardiopulmonary rehabilitation specialist nurses all have participated in cardiac rehabilitation training and obtained training certificates.

Patients were given continuous nursing care based on the hierarchical diagnosis and treatment mode. After discharge, patients were first admitted to a rehabilitation hospital for rehabilitation exercise, generally for 2–3 weeks. Then they were transferred to a community hospital for rehabilitation consolidation, according to the evaluation of nurses and doctors specializing in cardiac rehabilitation, to complete Stage II rehabilitation, as shown in Figure 2. The job responsibilities of the cardiopulmonary rehabilitation nursing specialist nurse are shown in Figure 3.

#### Evaluation indexes

Cardiac function indexes, quality of life, cardiac rehabilitation compliance, and medication compliance of patients in the two groups after PCI were collected at discharge and 6 months after discharge. The LVEF index can reflect myocardial ischemia and cardiac function, and is important for predicting the tolerability of PCI surgery. The 6MWD test is used to evaluate the exercise risk and prognosis of patients with heart disease, where 6MWD > 450 m is considered low risk, 6MWD 300–450 m is medium risk, 6MWD < 300 m is high risk, 6MWD < 150 m is extremely high risk, and longer walking distance indicated better cardiac function.

Quality of life was measured by the SF-36 Quality of Life Scale (SF36), which includes eight dimensions: physical functioning (PF), role physical (RP), bodily pain (BP), general health (GH), vitality (VT), social functioning, role emotional (RE), and mental health (MH), with 100 points for each dimension, where a higher score indicates a better quality of life.

The calculation method for cardiac rehabilitation compliance referred to the percentage of patients' actual/expected participation in Stage II cardiac rehabilitation, where a participation rate >50% was defined as high compliance.

The Morisky medication compliance scale was used to determine the medication compliance. The scale consists of four questions, which are mainly aimed at the conditions of medication and unauthorized drug withdrawal, and the answers to each question included “often,” “occasionally,” and “never,” corresponding to 1, 2, and 3 points, for a maximum of 12 points (4).

#### Statistical methods

IBM^®^ SPSS™ Statistics v21.0 software was used for statistical analysis. Measurement data were expressed as mean ± standard deviation ($\bar{x}$ ± s) and enumeration data were expressed as frequency and percentage. The chi-square test was used for comparison between the two groups. Those that did not obey the normal distribution were described by the median and interquartile range [M(Q)R], and the Kruskal-Wallis test was used for comparison between groups. One-way ANOVA was used to compare multiple groups, and paired t-test was used to compare before and after intervention. The differences were considered statistically significant if *P* < 0.05.

## Results

### Comparison of left ventricular ejection fraction among the three groups of patients after rehabilitation

Compared with the control group (48.04 ± 6.87), LVEF in the community group and the rehabilitation group was significantly improved after rehabilitation, which were 56.63 ± 8.03 and 63.61 ± 7.77, respectively, and the improvement in the rehabilitation group was more obvious. The differences were statistically significant (*P* < 0.01, Table 2).

**Table 2:** Comparison among three groups of patients in terms of LVEF before and after rehabilitation ($\bar{x}$ ± s).

**Group**	**Number of cases**	**LVEF**		**t**	***P***
		**Before rehabilitation**	**After rehabilitation**		
Control group	28	47.86 ± 5.18	48.04 ± 6.87	−1.088	0.286
Community group	30	49.20 ± 4.80	56.63 ± 8.03	−9.047	< 0.01
Rehabilitation group	33	48.20 ± 5.23	63.61 ± 7.77	−12.087	< 0.01
*F*		0.842	31.851		
*P*		0.434	< 0.01		

### Comparison of the 6-minute walking distance among the three groups of patients after rehabilitation

There was no significant improvement in 6MWD before and after rehabilitation in the control group (*P* > 0.05). However, the 6MWD of community group and rehabilitation group reached 373.47 ± 68.81 and 431.97 ± 73.39 respectively after rehabilitation, which was significantly higher than that of control group (310.14 ± 68.32), and was also significantly improved compared with the two groups before rehabilitation, with statistical significance (*P* < 0.01, Table 3).

**Table 3:** Comparison of 6MWD before and after rehabilitation among the three groups ($\bar{x}$ ± s).

**Group**	**Number of cases**	**6MWD**		**t**	***P***
		**Before rehabilitation**	**After rehabilitation**		
Control group	28	322.21 ± 50.75	310.14 ± 68.32	−0.755	0.457
Community group	30	317.3 ± 63.17	373.47 ± 68.81	3.55	0.001
Rehabilitation group	33	303.33 ± 61.31	431.97 ± 73.39	7.57	< 0.01
*F*		0.859	22.718		
*P*		0.427	< 0.01		

### Comparison of SF-36 scores between the three groups of patients after rehabilitation

Compared with the control group, the quality of life of the community group and rehabilitation group was significantly improved after rehabilitation. The eight dimensions of PF, RP, BP, GH, VT, RE, and MH in the community group and the rehabilitation group were significantly greater than those in control group, and the improvement in rehabilitation group was more obvious. The difference was statistically significant (*P* < 0.01, Table 4).

**Table 4:** Comparison among three groups of patients in terms of quality of life before and after rehabilitation ($\bar{x}$ ± s).

**Groups**	**Physiological function**		**t/*P***	**Physiological role**		**t/P**	**Body pain**		**t/*P***
	**Before rehabilitation**	**After rehabilitation**		**Before rehabilitation**	**After rehabilitation**		**Before rehabilitation**	**After rehabilitation**	
Control group	41.79 ± 3.51	46.64 ± 5.24	4.419/ < 0.05	44.96 ± 6.19	50.50 ± 5.12	−5.385/ < 0.05	49.71 ± 5.66	49.54 ± 5.58	0.108/0.914
Community group	42.13 ± 3.56	57.80 ± 4.98	−15.347/ < 0.01	45.83 ± 6.61	63.90 ± 6.09	−12.168/ < 0.01	48.80 ± 6.14	62.10 ± 4.62	−10.035/ < 0.01
Rehabilitation group	42.12 ± 4.08	64.94 ± 5.62	−19.644/ < 0.01	45.48 ± 6.19	61.30 ± 5.76	−20.14/ < 0.01	47.33 ± 5.58	70.30 ± 5.58	−15.32/ < 0.01
*F*	0.081	90.931		0.138	86.077		1.321	114.144	
*P*	0.923	< 0.01		0.871	< 0.01		0.272	< 0.01	
**Groups**	**Vitality**		**t/** ***P***	**Social function**		**t/** ***P***	**Emotional function**		**t/** ***P***
	**Before rehabilitation**	**After rehabilitation**		**Before rehabilitation**	**After rehabilitation**		**Before rehabilitation**	**After rehabilitation**	
Control group	44.75 ± 5.43	50.04 ± 5.69	−3.716/0.001	55.14 ± 10.82	60.92 ± 11.23	−2.372/0.025	52.32 ± 4.69	58.00 ± 6.46	−5.018/ < 0.05
Community group	46.13 ± 5.49	64.53 ± 5.08	−14.336/ < 0.01	60.23 ± 10.84	55.57 ± 10.27	1.709/ < 0.098	53.30 ± 4.69	67.33 ± 7.99	−7.633/ < 0.01
Rehabilitation group	44.03 ± 5.50	69.55 ± 5.59	−18.502/ < 0.01	54.58 ± 12.68	55.15 ± 10.18	−0.231/ < 0.819	52.03 ± 5.34	75.70 ± 6.76	−16.948/ < 0.01
*F*	1.186	102.054		2.601	2.725		0.559	46.985	
*P*	0.31	< 0.01		0.08	0.071		0.574	< 0.01	
**Groups**		**Mental health**		**t/** ***P***	**General health perceptions**		**t/** ***P***		
		**Before rehabilitation**	**After rehabilitation**		**Before rehabilitation**	**After rehabilitation**			
Control group		42.46 ± 4.00	61.00 ± 4.53	−15.853/ < 0.05	44.75 ± 4.90	57.29 ± 5.71	−9.018/ < 0.05		
Community group		44.03 ± 4.21	73.77 ± 7.75	−17.108/ < 0.01	45.13 ± 4.44	65.03 ± 4.66	−16.188/ < 0.01		
Rehabilitation group		46.12 ± 6.38	77.36 ± 7.09	−19.024/ < 0.01	44.45 ± 3.91	72.55 ± 5.62	−25.999/ < 0.01		
F		2.39	49.199		0.187	61.607			
*P*		0.098	< 0.01		0.83	< 0.01			

### Comparison of cardiac rehabilitation compliance between the two groups of patients

The data showed that the proportion of patients with cardiac rehabilitation compliance >50% in rehabilitation group was 48.5%, which was significantly higher than that in community group (6.7%), and the difference was statistically significant (*P* < 0.05, Table 5).

**Table 5:** Number of patients in compliance with cardiac rehabilitation (%).

**Groups**	**Number** **of cases**	**Compliance** **> 50%**	**Compliance** **< 50%**
Community group	30	2 (6.7)	28 (93.3)
Rehabilitation group	33	16 (48.5)	17 (51.5)
X^2^		13.465	
*P*		< 0.01	

### Comparison of medication compliance among the three groups

Morisky drug-taking scale recoveries were 100% in all three groups of patients. Compared with the control group, the community group and the rehabilitation group had significantly higher medication compliance, with the rehabilitation group more so. The differences were statistically significant (*P* < 0.01, Table 6).

**Table 6:** Comparison of patients' medication compliance.

**Groups**	**Number** **of cases**	**Medication** **compliance**
Control group	28	6.3 + 1.1
Community group	30	7.8 + 1.4
Rehabilitation group	33	10.9 + 1.1
*F*		121.416
*P*		< 0.01

Two continuous nursing care modes based on the hierarchical diagnosis and treatment mode have been proposed: tertiary hospital to community hospital, and tertiary hospital to professional rehabilitation hospital to community hospital. Compared with traditional cardiac rehabilitation, the two modes could significantly improve the patients' quality of life, LVEF, 6MWD, and medication compliance, with the differences observed in the present study being statistically significant (*P* < 0.05). The improvement in the rehabilitation group was more significant than that of the community group, with the differences being statistically significant (*P* < 0.05). The compliance with cardiac rehabilitation in the rehabilitation group was higher than that in the community group, with the difference being statistically significant (*P* < 0.05).

### Comparison of BMI of three groups of patients

The comparison of BMI before and after rehabilitation among the three groups shows that the BMI values of the community group and the rehabilitation group decreased after cardiac rehabilitation, and the difference was statistically significant at *P* < 0.05; but there was no significant difference between the three groups at *P* > 0.05.

## Discussion

### Continuous nursing cardiac rehabilitation mode based on hierarchical diagnosis and treatment can promote the recovery of patients' cardiac function

Specialist nurses play an indispensable role in cardiac rehabilitation (5); moreover, continuous nursing care has also been proven to improve the self-management ability of patients after PCI, and reduce the re-hospitalization rate, mortality rate, and medical cost (6). Hospital–community linkage continuing care is more common now and mainly refers to the combination of hospital and community nurses to extend nursing services to communities and families (7). As proposed in the 19th National Congress of the Communist Party of China, it is necessary to comprehensively develop a hierarchical medical system. In this study, hierarchical diagnosis and treatment was used for linkage between the upper and lower levels of hospitals, breaking the pattern of “difficult downward transfer,” so that the upper-level hospitals could teach the relevant rehabilitation nursing theories and skills to the lower-level hospitals, and the lower-level hospitals could report the problems to the higher-level hospitals, thus effectively linking all levels of hospitals to achieve the purpose of regional collaborative development. It not only can save the resources of tertiary general hospitals, but can also combine hospitals at all levels to achieve continuous nursing care with cardiac specialist nurses in the leading role.

The most widely accepted cardiac function rehabilitation index in clinical practice is LVEF (8), while 6MWD is one of the most common sub-extreme exercise tests, which can effectively assess the overall activity ability of patients and evaluate their cardiac functioning (9). According to the study results, the comparison before and after the application of continuous nursing cardiac rehabilitation mode based on the hierarchical diagnosis and treatment showed that the LVEF and 6MWD were not significantly improved in the observation group (*P* > 0.05), while the LVEF and 6MWD were significantly improved in the community group and the rehabilitation group (*P* < 0.05), and the improvement in the rehabilitation group was more significant than in the community group. Therefore, the continuous nursing cardiac rehabilitation mode based on hierarchical diagnosis and treatment was beneficial to the improvement of patients' cardiac functioning.

It has been proven that cardiac rehabilitation is beneficial to cardiac function recovery of patients after PCI. In the continuous nursing care mode based on hierarchical diagnosis and treatment, Stage II cardiac rehabilitation patients can be transferred to rehabilitation hospitals after PCI for professional rehabilitation exercise before being transferred to community hospitals. Continuous nursing care can be implemented during the entire time, which not only avoids the unbalanced development of cardiac rehabilitation in the community but also improves the quality of patients' cardiac rehabilitation by helping them to form beneficial habits in specialist hospitals.

### Continuous nursing cardiac rehabilitation mode based on hierarchical diagnosis and treatment can improve patients' quality of life

The SF36 has been widely used to evaluate the health status of patients with cardiovascular disease and can fully reflect their quality of life. In the present study, the SF36 was used to evaluate the patients' quality of life at discharge and 6 months after discharge, and showed that the scores for the seven factors PF, RP, BP, GH, VT, RE, and MH were improved after recovery, with the differences statistically significant (*P* < 0.05).

Many studies at home and abroad have shown that cardiac rehabilitation can improve the cardiopulmonary functioning of patients with CHD, reduce the occurrence of complications, and improve their quality of life (10, 11). The continuous nursing cardiac rehabilitation mode based on hierarchical diagnosis and treatment was shown to improve the patients' quality of life, and the study results were similar to those of other studies on cardiac rehabilitation in China.

Social functioning was improved in the observation group, but the level in the rehabilitation group and the community group was the same before and after rehabilitation, with no improvement found (*P* > 0.05). Sun et al. (12) used the SF36 for the comparison between patients with cardiovascular disease and the domestic norm, and found there were no differences between patients and non-patients in social functioning (*P* > 0.05), in which the score of the Chinese norm was 83 ± 17.8, and the score of the patients was 78.1 ± 29.4. In the present study, the score of patients before and after rehabilitation ranged from 50 to 70, which was lower than that in the study conducted by Sun et al. This might be related to the fact that patients in the rehabilitation group and the community group received rehabilitation more often, spent most of their time in hospitals, had a smaller social scope, and focused their time and energy on cardiac rehabilitation, or there might be some differences between local and domestic norms.

### Continuous nursing cardiac rehabilitation mode based on hierarchical diagnosis and treatment can improve patients' compliance with cardiac rehabilitation

The theory of cardiac rehabilitation was established in foreign countries as early as the 1950s, but a survey conducted in 22 European countries found that the participation rate of cardiac rehabilitation was only 36.5% despite a high level of recommendation (13). As shown in a British study, only 57.3% of patients undergoing cardiac rehabilitation completed the 8-week cardiac rehabilitation course as required (7). The compliance of cardiac rehabilitation in patients after PCI was only 6% (14), and the rehabilitation referral rate was only 48% (15).

In the present study, among the patients undergoing Stage II cardiac rehabilitation who participated for 6 months, 16 patients (48.5%) in the rehabilitation group showed a high rehabilitation compliance, and 9 patients (27.3%) followed the original rehabilitation plan exactly; 2 patients (6.7%) in the community group showed a high rehabilitation compliance, and 0 patients followed the original rehabilitation plan exactly. The comparison between the two groups showed that the rehabilitation group had a higher rehabilitation compliance than the community group (*P* < 0.05). Rehabilitation compliance ≤ 50% is considered as low compliance by foreign scholars (16). Although patients' compliance with cardiac rehabilitation and their cardiac function were improved in the experimental group to a certain extent, the overall compliance level was still lower than the level in foreign countries. This might be related to the fact that the domestic medical insurance system is imperfect, the medical cost of rehabilitation is high, and the acceptance level of cardiac rehabilitation among the people is low. The mortality within 10 years after PCI is up to 30%, and the proportion of patients who experience angina within 1 year after PCI is up to 32.3% (17, 18). Therefore, it is imperative to find an effective path and mode of Stage II cardiac rehabilitation and improve the health of patients after PCI.

### Continuous nursing cardiac rehabilitation mode based on hierarchical diagnosis and treatment can improve the medication compliance of patients

Dual antiplatelet drugs should be taken for 6 months to 1 year after PCI to greatly reduce the risk of thrombosis, re-infarction, and death. Nevertheless, studies have shown (19) that the medication compliance of patients with chronic diseases is still not optimistic, and 40–75% of elderly patients with chronic diseases cannot follow the prescribed medication (20, 21). Patients with good medication compliance can obtain better efficacy and reduce the incidence of complications. According to the results of the present study, patients in the two continuous nursing cardiac rehabilitation modes based on hierarchical diagnosis and treatment showed a higher medication compliance than the observation group, and the medication compliance in the rehabilitation group was higher than in the community group, with a score of 10.9 + 1.1. In the continuous nursing cardiac rehabilitation mode based on hierarchical diagnosis and treatment, patients receive more health education on drugs and diseases from specialist cardiac nurses while doing rehabilitation exercise, thus improving the importance patients attach to taking medication.

### Continuous nursing cardiac rehabilitation mode based on hierarchical diagnosis and treatment can reduce the BMI value of patients to a certain extent

Studies have shown (22) that people with a BMI ≥ 28.0 kg/m^2^ have twice the risk of developing the disease than those with a normal BMI, and BMI has a similar predictive value for coronary heart disease (23). BMI may be a good indicator of cardiometabolic risk (24). Chinese people have the habit of self-cultivation after being ill. Although cardiac rehabilitation has been continuously implemented in China in recent years, middle-aged and elderly people still cannot accept the concept of cardiac rehabilitation very well. This study found that the BMI value of the observation group did not change during the entire trial; the BMI value of the patients after cardiac rehabilitation intervention was lower than that before the intervention (Table 7). It may be related to cardiac rehabilitation, which can urge patients to exercise rehabilitation and healthy diet education, so that patients who are overweight can lose weight to a certain extent, which is beneficial to their weight management and health. However, due to the small amount of data collected, further research is needed on whether the continuous care cardiac rehabilitation model based on the hierarchical diagnosis and treatment model can effectively reduce the BMI value of patients.

**Table 7:** Comparison of BMI of three groups of patients before and after rehabilitation.

**Group**	**Number of cases**	**BMI**		**t**	***P***
		**Before rehabilitation**	**After rehabilitation**		
Control group	28	25.49 ± 1.44	25.41 ± 1.31	1.276	0.213
Community group	30	25.57 ± 1.59	24.77 ± 1.49	5.446	< 0.01
Rehabilitation group	33	26.22 ± 1.49	24.78 ± 1.22	10.804	< 0.01
F		2.256	2.156		
P		0.111	0.122		

BMI, body mass index.

In conclusion, the continuous nursing care mode based on hierarchical diagnosis and treatment is beneficial to the Stage II cardiac rehabilitation of patients after PCI, and it is more advantageous by means of the path of tertiary hospital to professional rehabilitation hospital to community hospital. There are still deficiencies in this study due to the small sample size. Follow-up multi-center studies will be attempted to further validate the effect of applying continuous nursing care based on hierarchical diagnosis and treatment in Stage II cardiac rehabilitation of patients after PCI.

## Data availability statement

The original contributions presented in the study are included in the article/supplementary material, further inquiries can be directed to the corresponding author.

## Ethics statement

The studies involving human participants were reviewed and approved by Ethics Committee of Shanghai Songjiang District Central Hospital (The First People's Hospital of Shanghai Jiao Tong University, Songjiang Branch) (Approval Number: 2020SQ020). The patients/participants provided their written informed consent to participate in this study.

## Author contributions

Conception and design of the research: Z-JD and J-YZ. Acquisition of data: P-HG and S-TX. Analysis and interpretation of the data: J-FZ. Statistical analysis: S-TX and C-FZ. Obtaining financing: Z-JD. Writing of the manuscript: Z-JD and S-TX. Critical revision of the manuscript for intellectual content: J-YZ. All authors read and approved the final draft.

## Funding

This work was supported by Scientific Research Project of Shanghai Nursing Society (No. 2020MS-B09).

## Conflict of interest

The authors declare that the research was conducted in the absence of any commercial or financial relationships that could be construed as a potential conflict of interest.

## Publisher's note

All claims expressed in this article are solely those of the authors and do not necessarily represent those of their affiliated organizations, or those of the publisher, the editors and the reviewers. Any product that may be evaluated in this article, or claim that may be made by its manufacturer, is not guaranteed or endorsed by the publisher.
